# Impact of exercise on cognitive efficiency in older adults with subjective memory complaints: a randomized clinical trial

**DOI:** 10.3389/fnagi.2026.1875431

**Published:** 2026-08-05

**Authors:** Gustavo O. Silva, Melanie Clark, Nicola T. Lautenschlager, Hannah J. Thomas, Kurt J. Smith, Barbara A. Maslen, Kay L. Cox, Carmela F. Pestell, Philip N. Ainslie, Howard H. Carter, Louise H. Naylor, Daniel J. Green

**Affiliations:** 1 School of Human Sciences (Exercise and Sport Science), The University of Western Australia, Perth, WA, Australia; 2 Faculdade Pernambucana de Saúde, Recife, PE, Brazil; 3 Neurosciences Institute, Perron Institute, Nedlands, WA, Australia; 4 Department of Psychiatry, The University of Melbourne, Parkville, VIC, Australia; 5 Older Adult Mental Health Program, The Royal Melbourne Hospital, Melbourne, VIC, Australia; 6 Centre for Healthy Ageing, Health Futures Institute, Murdoch University, Murdoch, WA, Australia; 7 Cerebrovascular Health, Exercise, and Environmental Research Sciences Laboratory, School of Exercise Science and Physical Health Education, University of Victoria, Victoria, BC, Canada; 8 Medical School, University of Western Australia, Perth, WA, Australia; 9 School of Psychological Science, University of Western Australia, Perth, WA, Australia; 10 Centre for Heart, Lung and Vascular Health, School of Health, and Exercise Science, University of British Columbia, Kelowna, BC, Canada

**Keywords:** cerebral blood flow, cerebrovascular function, cognition, cognitive ageing, exercise

## Abstract

**Introduction:**

Cerebrovascular dysfunction may contribute to cognitive impairment in older adults. Exercise is recommended for preventing cerebrovascular health and cognition; however, it remains unclear whether cognitive changes are related to changes in cerebrovascular function. Thus, this study aimed to examine whether exercise-induced changes in cognition and subjective memory complaints are associated with changes in cerebral blood velocity.

**Methods:**

This study is a randomized controlled trial that included 72 sedentary older adults with memory complaints (age = 62 ± 7, 19 [26%♂]). Participants were randomized to a land-walking (LW), water-walking (WW), or control group (C). Exercise groups (*n* = 49, age = 63 ± 6, 13 [27%♂]) completed 24 weeks of supervised training (3 times/week, 50 min/session, at 40–65% of heart rate reserve). Cognitive outcomes included the Repeatable Battery for the Assessment of Neuropsychological Status (RBANS) and Trail Making Test (TMT), and subjective memory was assessed using the Memory Complaint Questionnaire (MAC-Q). Middle (MCAv) and posterior (PCAv) cerebral artery blood velocity was assessed at rest and during visual neurovascular coupling, hypercapnic reactivity, and cerebral autoregulation.

**Results:**

TMT-B improved in the WW group compared to C (*p* = 0.031). In pooled exercise groups, improvements in RBANS (*r* = −0.386, *p* = 0.024) and MAC-Q (*r* = 0.357, *p* = 0.035) were associated with reduced resting MCAv. No significant associations were observed for other cerebrovascular measures.

**Conclusion:**

Improvements in neuropsychological status and subjective memory were associated with reduced resting MCAv following exercise training. These exploratory associations suggest that variability in cerebrovascular responses may relate to cognitive responses; however, without consistent group-level effects, findings should be interpreted cautiously and do not imply causality.

**Clinical trials registration:**

https://www.anzctr.org.au/, ACTRN12614000017628.

## 1 Introduction

As population life expectancy increases (Abbafati et al., 2020), an ongoing challenge is to maintain brain health. Ageing can be associated with reduced cognitive function (Murman, 2015), and dementia has rapidly increased in prevalence (Nichols et al., 2022). It is estimated that the global burden of dementia may increase from 57.4 million cases in 2019 to 152.8 million by 2050 (Nichols et al., 2022). The contribution of dementia, as a share of global healthcare costs, increased by 4.5% from 2000 to 2019, and this is projected to reach 11% by 2050 (Velandia et al., 2022).

Cerebral blood flow can also be impaired with ageing (Mokhber et al., 2021), and cerebrovascular dysfunction may contribute to deterioration in brain health in older adults (Bangen et al., 2014). Age-related changes in cerebrovascular function may be associated with microvascular disease, ischaemic stroke, and the early stages of dementia (Raz et al., 2016). Whilst pharmacological strategies to preserve brain health, have shown limited and variable efficacy, regular aerobic and resistance exercise are among the most effective non-pharmacological strategies to maintain or attenuate age-related declines in cognitive function (Lautenschlager et al., 2010). A possible mechanism for improvements in cognition with exercise is its known beneficial effects on vascular function and endothelial health (Barnes and Corkery, 2018; Green and Smith, 2018; Song et al., 2025).

Vascular adaptations to systemic stimuli may be vessel bed-specific in humans (Carter et al., 2017), highlighting the need to target exercise interventions to specific vascular networks. Increasing shear stress within the cerebrovasculature during exercise may therefore have a greater impact on vascular function and cognition. Water immersion has been shown by our group to increase cerebral blood velocity both at rest and during exercise (Carter et al., 2014; Pugh et al., 2015), enhancing cerebrovascular shear stress and representing a potential strategy to improve cognitive health.

Recent evidence supports a close relationship between cerebrovascular function and cognitive performance in the context of exercise interventions. For example, aerobic exercise training over 6 months has been shown to improve both cerebrovascular function and cognitive outcomes, with evidence of an association between these adaptations (Guadagni et al., 2020). In addition, observational studies indicate that individuals with neurogenerative disease or cognitive impairment often exhibit reduced cerebrovascular function (Wolters et al., 2017; Fresnais et al., 2023). Together, these findings support a potential mechanistic link between cerebrovascular health and cognitive function. However, relatively few studies have directly examined whether exercise-induced changes in cerebrovascular function are associated with concurrent changes in cognitive performance, particularly in otherwise healthy older adults with subjective memory complaints. This population may represent an early stage of cognitive vulnerability, in which interventions such as exercise could be more effective prior to the onset of irreversible structural or neurodegenerative changes.

Thus, we hypothesised that improvements in Neuropsychological Status (RBANS), executive function (Trail Making Test), and subjective memory (Memory Complaint Questionnaire [MAC-Q]), would relate to changes in middle cerebral artery velocity (MCAv) in older adults following supervised centre-based exercise programs. These analyses were undertaken to directly test this *a priori* hypothesis within the framework of the PREVENTIA trial (Green et al., 2018), with the primary aim of examining the effects of exercise training on cognitive outcomes, and secondary analyses assessing their relationship with previously reported cerebrovascular measures.

## 2 Materials and methods

### 2.1 Sample and study design

Healthy, sedentary men and postmenopausal women >50 yr. with subjective memory complaints were recruited via community-based strategies, including radio appeals, newspaper advertisements, and outreach to local community and aged groups, to participate in a 24-week randomized controlled intervention study (Green et al., 2018). Postmenopausal women were specifically included as part of a cognately healthy but at-risk ageing cohort characterised by subjective memory complaints and increased susceptibility to age-related cognitive and cerebrovascular decline, thereby enabling investigation of exercise-induced adaptations in a clinically relevant pre-dementia population. Participants were screened using structured telephone interviews and validated instruments (e.g., Modified Telephone Interview Cognitive Status score [TICS-m], Standardized Mini-Mental State Examination [sMMSE], Geriatric Depression Scale [GDS], and RBANS) to confirm the absence of cognitive impairment and depression, alongside medical screening to exclude major cardiovascular, metabolic, and neurological conditions and ensure safe participation in exercise.

This study was conducted as part of the PREVENTIA randomised controlled trial, which included a pre-specified hypothesis that improvements in cerebrovascular function would be associated with changes in cognition, memory, and clinical outcomes (hypothesis 3) (Green et al., 2018). While cerebrovascular outcomes from this study have been reported previously, including detailed methodological descriptions and primary cerebrovascular findings (Green et al., 2021), the present study incorporates newly analysed cognitive outcomes and specifically examines their relationship with cerebrovascular measures within the randomised controlled trial framework. Objective cognition was assessed using the RBANS and Trail Making Test, while subjective memory was assessed using the MAC-Q, and these measures were considered complementary rather than hierarchical outcomes within the context of this analysis.

### 2.2 Standard protocol approvals, registrations, and patient consents

This study was approved by The University of Western Australia’s Human Research Ethics Committee, that conformed to the standards set by the Declaration of Helsinki. All participants provided written, informed consent before any assessments being performed.

### 2.3 Inclusion and exclusion criteria

Exclusion criteria were diagnosed mild cognitive impairment or dementia (based on prior clinical diagnosis or objective screening at baseline), smoking (or <12-month cessation), hypertension (systolic blood pressure >160 mmHg or diastolic blood pressure >100 mmHg), weekly alcohol intake greater than 280 g^.^wk.^−1^ and/or drinking more than 40 g ethanol in one session, body mass index >40 kg^.^m^−2^, and total cholesterol greater than 7.0 mmol L^−1^. Inclusion criteria were initially assessed via a structured phone interview and subsequently confirmed during baseline laboratory screening. Participants were required to be 50 years or older (women were postmenopausal), healthy but inactive – less than 60 min wk.^−1^ of moderate or higher intensity exercise. The baseline screening visit included blood tests and a resting electrocardiogram (ECG) and 12-lead ECG stress test. Blood samples were analysed for standard clinical biochemistry, including lipids, glucose, renal function, and haematological parameters. ECG recordings (resting and during exercise) were assessed by medical practitioners to screen for cardiovascular abnormalities, and any findings of concern were referred to the participant’s general practitioner, with exclusion from the study or further evaluation undertaken as appropriate.

To rule out cognitive impairment, a battery of cognitive tests was administered by a research psychologist (under supervision of a qualified neuropsychologist) in the screening stages (with formal cognitive assessment conducted at the baseline laboratory visit, following initial eligibility screening by phone interview). Participants were excluded if they had *a priori* objective cognitive impairment (mild cognitive impairment or dementia) or fulfilled one of the following categories: TICS-m score <32, GDS score >6, sMMSE score <24, or RBANS delayed memory index score more than 1.5 standard deviations below the age-related mean. Eligible participants were then randomised to a land-walking (LW), water-walking (WW), or usual care control group (C). The study outcomes (i.e., cognition and cerebrovascular function) were assessed in all participants at weeks 0 and 24.

### 2.4 Interventions

Participants allocated to the exercise training groups attended a research centre 3 times/week for 50 min supervised sessions, across 24 weeks (Green et al., 2018). Intensity of exercise was monitored with a heart rate watch (Polar RS300X HR monitor; Polar Electro Oy, Espoo, Finland). A supervised warm-up and cool-down interval was included in each session. Land-based walking took place on the University grounds as well as along the adjoining river foreshore and parkland, or on a treadmill if weather indicated. Water-based walking was in a heated (~30 °C) chest deep swimming pool (20 × 30 m) located at the University. Duration and intensity of the exercise sessions increased over the course of the study, from an initial 15 min of exercise, increasing to 50 min by the end of the 24 wk. Participants (land and water) were given a personalised target heart rate range, based on heart rate reserve calculated from measured peak heart rate obtained during a maximal exercise test. Heart rate reserve was used to prescribe exercise intensity in both land- and water-based groups, as it accounts for inter-individual variability and is considered appropriate for standardising exercise intensity across different exercise modalities, including water-based exercise where hydrostatic pressure may influence absolute heart rate responses. Each exercise session was guided by an accredited exercise scientist/physiologist (Exercise and Sports Science Australia), and heart rate was monitored and recorded every 5 min throughout the session. The second session of each week was an interval training session, with the other two sessions of continuous intensity. The inclusion of interval training was intended to provide periodic higher-intensity stimuli within a predominantly moderate-intensity walking program, in accordance with established exercise prescription guidelines, to optimise cardiovascular and vascular adaptations while maintaining feasibility in a previously sedentary population. Interval sessions consisted of alternating periods of higher and lower intensity walking prescribed withing the target heart rate reserve range, with progression over time; interval duration was not fixed but adjusted within sessions to maintain the prescribed intensity. The intensity of training started at 40–45% heart rate reserve, building to 55–65% by week 24. Additional, “make-up” sessions were provided if required, to ensure optimal adherence to the protocol. Adherence and training intensity (percentage of heart rate reserve) were quantified as previously described, with participants exercising within the prescribed intensity ranges. Mean attendance rates were 83.2 ± 4.7% for the LW group and 79.2 ± 4.8% for the WW group, with no significant differences in achieved exercise intensity between groups, with mean training intensity (expressed as %HRR) within the prescribed range (~40 to 65% HRR) across the intervention, and all participants training within the prescribed heart rate reserve ranges (Green et al., 2018; Haynes et al., 2020; Naylor et al., 2020).

The control group was asked to maintain their usual levels of activity (all participants were sedentary at recruitment) and attended the University every 6 weeks to participate in an educational seminar of approximately 1 h, on a topic that was expected to be of interest but was unrelated to physical activity, lifestyle, and health: for example, first aid skills, and computer workstation optimal ergonomic setup.

### 2.5 Primary outcome—cognition

The cognitive tests were conducted by a research psychologist, who was blinded to the allocation group. A second research psychologist independently scored all test results a second time to ensure accuracy and consistency. All testing was performed in a quiet room away from the laboratory and was completed in approximately 1 h with the tests order randomised.

The sMMSE is a brief cognitive test with a maximum score of 30. The test is widely used by physicians and as a screening test for cognitive impairment. Items on the sMMSE assess word recall, orientation to time and place, attention, language, and visuospatial abilities (Molloy and Standish, 1997).

The RBANS is a brief, reliable battery developed to measure cognitive decline or improvement in five domains of cognitive function: immediate and delayed memory, attention, language, and visuo-spatial skills. Specific tests capture episodic memory (list learning, story recall, and figure recall), speed of information processing (digit symbol coding), language abilities (picture naming and semantic fluency), visuo-construction skills (figure copy and line orientation), and attention (digit span). While some RBANS subtests may engage aspects of executive processing, the battery is not designed as a primary measure of executive function. Subdomains are scored to obtain a composite Total Score. The RBANS is suitable for use in adults <89 yr., is sensitive to a wide range of cognitive abilities, and is frequently used in clinical trials of treatments that impact neurocognitive status (Randolph et al., 1998). We used parallel RBANS Forms A and B across the two testing sessions to minimise practice effects.

The Trail Making Test (TMT) Parts A + B measure executive functioning abilities related to visual search, scanning, speed of processing, and mental flexibility. It is comprised of two parts: TMT-A requires participants to draw lines in the correct sequence connecting 25 encircled numbers, distributed on a sheet of paper. The TMT-B is similar, but participants must alternate between numbers and letters. Scoring is based on the amount of time it takes to complete each task, with longer completion times more indicative of impairment (Tombaugh, 2004).

The MAC-Q provides a brief assessment of subjective memory complaints. It is comprised of five items assessing memory in specific situations and one item measuring overall self-perceived memory decline. Respondents can answer either yes or no, and the total score ranges from 7 to 35. Drawing on earlier studies, a score of 25 and above on the MAC-Q is taken to be indicative of significant memory complaint, potentially requiring further assessment (Crook et al., 1992).

The Hospital Anxiety and Depression Scale (HADS) is a widely used measure of psychological distress, designed for use in non-psychiatric patient populations. The HADS consists of two 7-item subscales, measuring anxiety and depression respectively; though latent structure analysis suggests it is best used as a more general measure of distress (Cosco et al., 2012).

The WHO-5 WellBeing Index is among the most widely used measures of assessment of subjective psychological wellbeing. It uses a generic global rating scale, which only contains positively phrased items. Participants are asked to rate how well each of the 5 statements applies to them when considering the last 14 days, on a scale from 5 (all the time) to 0 (none of the time). The raw score ranges from 0 (lack of wellbeing) to 25 (maximal wellbeing) (World Health Organization, 1998).

### 2.6 Secondary outcome—cerebrovascular function

Cerebrovascular function was assessed on non-exercise days, at the same time of day, and the participants were asked to avoid moderate/vigorous physical activity, caffeine, and food for at least 6 h before the test. Middle (MCAv) and posterior (PCAv) cerebral artery blood velocities were measured using a 2-MHz, S3 transcranial Doppler (TCD) ultrasound system (Spencer Technologies, Seattle, WA). Cerebrovascular assessments followed previously published and validated protocols (Green et al., 2021), and adhered to established methodological guidelines (Willie et al., 2011). The procedures are briefly described below. During all assessments, MCAv, PCAv, and end-tidal CO_2_ (P_ET_CO_2_) were continuously recorded along with beat-to-beat blood pressure via photoplethysmography (Finometer; Finapres Medical Systems, Amsterdam, the Netherlands) and subsequently analysed offline. After instrumentation and a 20-min supine rest, a 5-min baseline recording was performed, from which resting MCAv and PCAv were derived as the mean values averaged across this period.

For the visual neurovascular coupling (NVC) measurement, a 4-min baseline was measured (2-min eyes open, 2-min eyes closed), followed by 5 cycles of 30 s eyes open and 30 s eyes closed. During the eyes open cycles, a tablet was placed in front of the participants’ eyes, and they were instructed to watch a 0.5-Hz chequerboard visual stimulus. The visual NVC response was calculated as the average peak %PCAv change across the five trials. The last 10 s of eyes closed was averaged and used as the baseline for the following peak PCAv during eyes open (Phillips et al., 2016).

For the cerebrovascular CO_2_ reactivity measurement, after a 5-min rest period breathing room air via a two-way mouthpiece, participants inhaled 3% CO_2_ for 4 min and then 6% CO_2_ for 4 min (both 21% O_2,_ balanced N_2_) via a Douglas bag. The MCAv and cerebrovascular conductance (CVC; calculated as MCAv/mean arterial pressure [MAP]) response to elevations in P_ET_CO_2_ was analysed by averaging 30s of data at baseline and during the last 30s of each hypercapnic stimulus. Linear regression analysis was then performed to calculate reactivity slopes (P_ET_CO_2_ vs. MCAv and CVC).

To measure dynamic cerebral autoregulation, blood pressure oscillations were induced by a lower-body negative pressure chamber (to −70 mmHg) (Vacustyler; Weyergans, Dueren, Germany) across a 4-min period of 10-s on/10-s off (0.05 Hz). Transfer function analysis (TFA) was performed using the commercially available software Ensemble-R (Elucimed Ltd., Wellington, New Zealand) that includes internationally recommended guidelines for autoregulation analysis (Claassen et al., 2016).

### 2.7 Statistics

Statistical analyses were conducted using SPSS version 23.0 (IBM Corporation, Armonk, NY) and STATA version 15.0 (STATA Corporation LP, College Station, TX). Unadjusted means, SD, and confidence intervals were calculated for the cardiovascular risk factor and vascular function outcome variables at weeks 0 and 24.

*A priori* sample size estimation was based on published data from Akazawa et al. (2012), which reported an increase in cerebral blood velocity (the primary outcome used for powering the study) from 62 ± 12 cm/s to 70 ± 12 cm/s following an exercise intervention. Assuming a similar intervention effect, with *α* = 0.05 and 80% power (calculated using G*Power), a minimum sample size of 20 participants was required to detect a statistically significant change in the primary outcome (cerebrovascular function). The present analysis of cognitive outcomes was not specifically powered and should therefore be interpreted as exploratory.

Due to the repeated-measure nature of the data, separate linear mixed models were used to investigate the relationship between the cognitive factors, group (C, LW, and WW), and time (weeks 0 and 24) using two-tailed tests. These analyses accounted for time-invariant covariates, including age and sex, and an interaction between group and time. A random intercept was included in each model to account for the repeated nature of the data. To maximise power for some analyses, we pooled the training groups and performed regression analyses, adjusted for age and sex. Statistical significance was set at *p* < 0.05.

Analyses were interpreted within a hierarchical framework, with cerebrovascular function defined as the primary outcome and cognitive and additional vascular outcomes considered secondary/exploratory; therefore, no formal adjustment for multiple comparisons was applied. Correlational analyses were conducted to evaluate *a priori* hypotheses specified in the PREVENTIA study protocol and should be interpreted as exploratory.

A full consort diagram, including the recruitment and screening process, along with details on sample size calculation, randomization, and allocation, is presented in the associated methodology paper (Green et al., 2018).

## 3 Results

**Figure 1:**
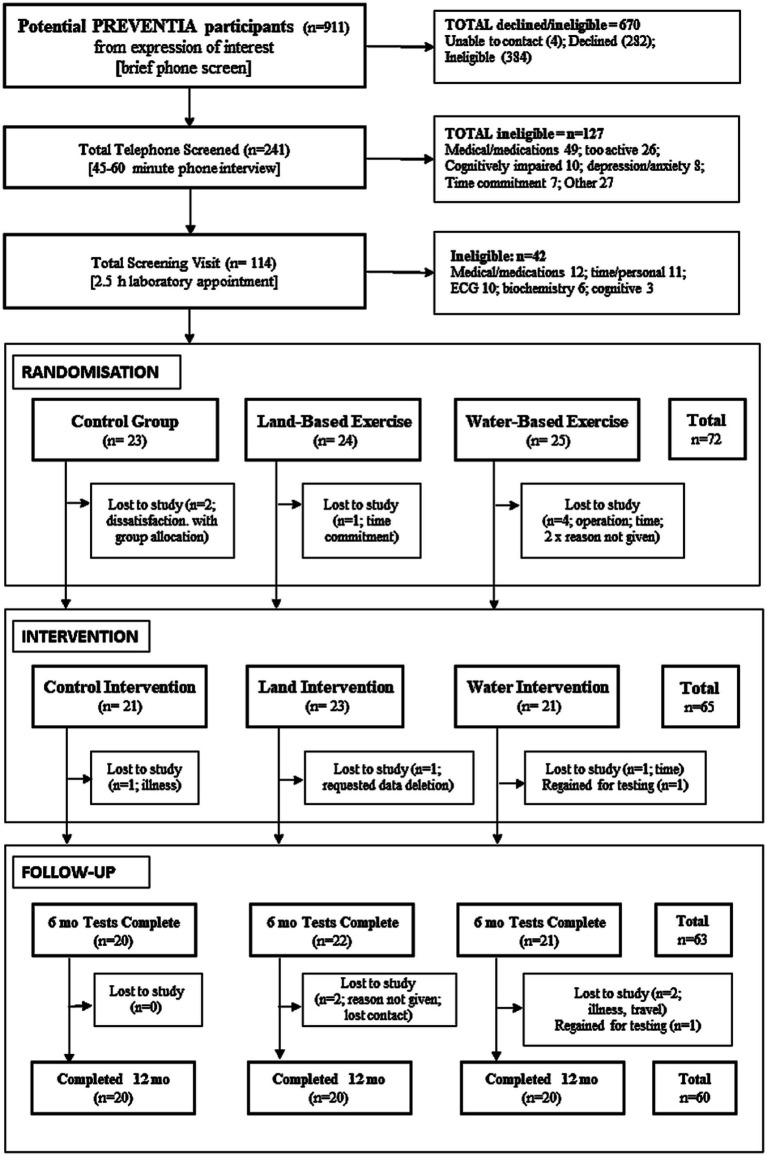
Consort diagram of enrolment into the study.

Full enrolment into the study for every screening step is present in the Consort diagram in Figure 1.

After the final screening, 72 participants were recruited and randomised into the study, as detailed elsewhere (Green et al., 2018). Baseline characteristics of the 72 participants included are presented in Table 1. Peak exercise test results have been reported previously (Haynes et al., 2020). Briefly, baseline cardiorespiratory fitness (VO_2_peak) did not differ between groups (*p* > 0.05), indicating comparable fitness status at study entry. Exercise training resulted in improvements in VO_2_ peak in both intervention groups (LW: 28.4 ± 7.85 mL.kg_-1_.min_-1_ Baseline vs. 29.5 ± 6.71 mL.kg_-1_.min_-1_ Week 24; WW: 28.8 ± 5.05 mL.kg_-1_.min_-1_ Baseline vs. 30.1 ± 5.30 mL.kg_-1_.min_-1_ Week 24), whereas no change was observed in the control group (29.7 ± 4.41 mL.kg_-1_.min_-1_ Baseline vs. 28.0 ± 3.02 mL.kg_-1_.min_-1_ Week 24; *p* < 0.05), confirming that the prescribed exercise stimulus was sufficient to elicit cardiorespiratory adaptations. Changes in body composition and cardiovascular risk factors from this cohort have also been reported previously (Naylor et al., 2020; Green et al., 2024). Briefly, exercise training induced favourable changes in selected body composition (i.e., central adiposity and lower limb lean mass), and cardiovascular health measures (i.e., LDL and total cholesterol/HDL ratio), whereas body mass and BMI did not change significantly between groups (*p* > 0.05). Regarding data availability, cognition or cerebral data were not available in nine participants, either because of withdrawal from the study or poor transcranial Doppler signal quality. Therefore, 63 of the 72 participants (13 [27%♂]) were included in the regression analyses. Differences in sample sizes between the present analysis and previously published cerebrovascular outcomes (Green et al., 2021) reflect the inclusion of participants with complete and usable data for the specific variables of interest, with the current analyses restricted to those with both cognitive and cerebrovascular data. No adverse events were reported during the intervention period.

**Table 1:** Baseline characteristics of the control, land walking and water walking groups.

Variable	Control group	Land walking	Water walking
n, M/F	*n* = 23; 6M, 17F	*n* = 24; 6M, 18F	*n* = 25; 7M, 18F
Age, yr	62 ± 7	63 ± 7	63 ± 7
Height, cm	168 ± 10	165 ± 8	167 ± 7
Weight, kg	73.8 ± 13.6	74.4 ± 11.1	76.8 ± 19.8
BMI, kg·m^−2^	26.2 ± 4.1	27.3 ± 3.5	27.3 ± 5.6
Education level, yr	15 ± 3	15 ± 3	16 ± 4

Values are presented as mean ± SD. There were no significant baseline differences between the groups. BMI, body mass index; F, female; M, male.

The effects of the interventions on cognition measures are presented in Table 2. Compared to the control group, there were no interaction effects in cognitive domains or memory from weeks 0 to 24 (*p* > 0.05 for all), except for TMT-B, in which the WW group showed a greater improvement compared to the control group (Group × Time interaction, *p* = 0.031), but the same did not occur for the LW group in comparison to the control group (Group × Time interaction, *p* = 0.079). Data on cerebrovascular outcomes have been published elsewhere (Green et al., 2021). Briefly, the results demonstrated no systemic changes across different domains of cerebrovascular function with exercise training (i.e., MCAv and PCAv at rest and in response to visual NVC, hypercapnic reactivity, and cerebral autoregulation). As reported previously, these cerebrovascular outcomes are not interpreted as primary outcomes in the present manuscript. The focus of the present paper was the relationship between these previously published cerebrovascular variables and cognition measures.

**Table 2:** Cognition before and after the 24-week intervention.

Variable	Control group		Land walking		Water walking	
	Week 0	Week 24	Week 0	Week 24	Week 0	Week 24
sMMSE, score	28 ± 1	28 ± 1	29 ± 1	28 ± 1	28 ± 1	28 ± 1
RBANS (score)						
Delayed memory^†^	101 ± 10	107 ± 9	106 ± 8	109 ± 10	102 ± 10	108 ± 8
Immediate memory	100 ± 16	103 ± 14	100 ± 11	104 ± 12	92 ± 13	100 ± 10
Visuospatial	111 ± 15	109 ± 12	116 ± 11	110 ± 15	106 ± 16	111 ± 15
Language^*^^,^^†^	102 ± 8	106 ± 10	107 ± 11	109 ± 10	103 ± 9	102 ± 12
Attention	105 ± 12	103 ± 16	105 ± 13	106 ± 17	106 ± 11	108 ± 15
Total scale^*^	105 ± 11	108 ± 10	110 ± 12	111 ± 14	102 ± 10	108 ± 10
Total index	518 ± 36	527 ± 36	534 ± 37	538 ± 42	509 ± 34	529 ± 34
Trail making test part A (sec)	34 ± 8	31 ± 9	33 ± 10	32 ± 9	29 ± 7	30 ± 9
Trail making test part B (sec)^‡^	66 ± 21	67 ± 24	68 ± 28	60 ± 21	76 ± 27	66 ± 18
HADS anxiety (score)	5 ± 3	5 ± 3	5 ± 2	5 ± 3	4 ± 3	4 ± 3
HADS depression (score)	3 ± 2	4 ± 2	2 ± 1	2 ± 1	3 ± 2	3 ± 4
HADS total (score)	8 ± 4	9 ± 5	7 ± 3	6 ± 4	6 ± 5	7 ± 6
WHO-5 wellbeing index (score)	17 ± 3	16 ± 4	17 ± 3	18 ± 3	18 ± 3	17 ± 4
MAC-Q (score)^*^	24 ± 3	24 ± 3	22 ± 4	21 ± 4	23 ± 3	22 ± 4

Results are presented as mean ± standard deviation. HADS, hospital anxiety and depression scale; MAC-Q, memory complaint questionnaire; sMMSE, standardized mini-mental state examination; RBANS, repeatable battery for the assessment of neuropsychological status; WHO, world health organization; LW, land walking; WW, water walking; C, control.

* Significant group effect.

† Significant time effect.

‡ Significant interaction effect (WW vs. C).

**Figure 2:**
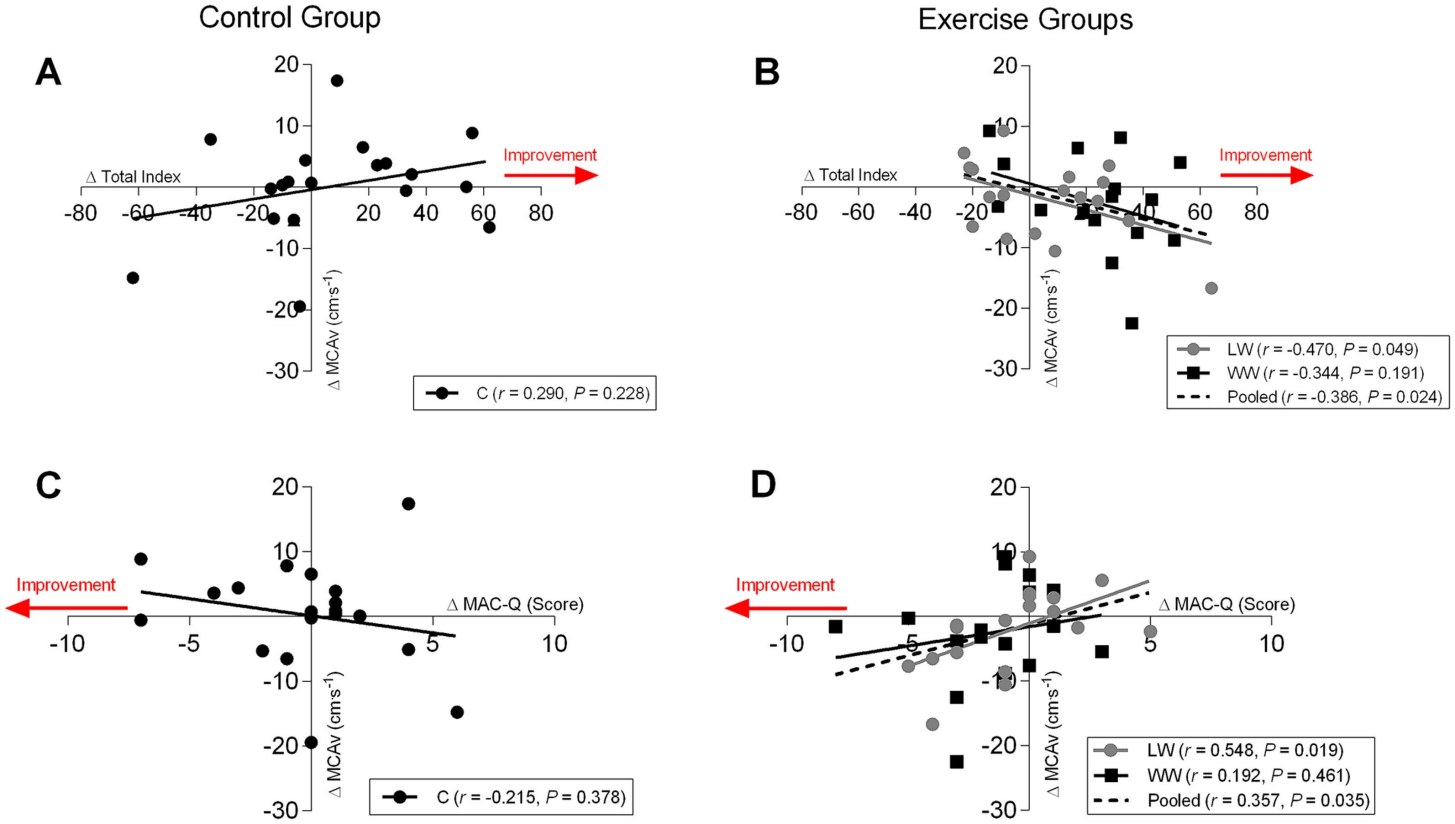
Correlation between changes in the RBANS total index, MAC-Q scores, and changes in resting MCAv linear regressions from weeks 0 to 24. There was a significant negative correlation for changes in the RBANS total index in the exercise groups **(B)**, whereby a larger increase in the RBANS total index was associated with a lower resting MCAv, but the same did not occur with the control group **(A)**. Also, there was a significant positive correlation for changes in MAC-Q scores in the exercise groups **(D)**, whereby a larger decrease in MAC-Q scores was associated with a lower resting MCAv, which was not the case for the control group **(C)**. C, control; LW, land walking; WW, water walking. MAC-Q, Memory Complaint Questionnaire RBANS, Repeatable Battery for the Assessment of Neuropsychological Status MCAv, middle cerebral artery velocity.

We plotted change in cerebrovascular outcomes against changes in cognitive outcomes and subjective memory complaints between weeks 0 and 24 in the pooled exercise groups and in the control group. Results showed that improvement in RBANS with training was significantly correlated with decreased resting MCAv in the exercise groups (*r* = −0.386, *p =* 0.024; Figure 2B), but not in the controls (*r* = 0.290, *p =* 0.228; Figure 2A). Improvement in MAC-Q with training was correlated with a decrease in resting MCAv in the exercise groups (*r* = 0.357, *p =* 0.035; Figure 2D), but not the control participants (*r* = −0.215, *p =* 0.378; Figure 2C), indicating that greater improvements in cognition and memory were associated with a decrease in resting brain blood velocity. No significant association was observed between changes in TMT-B and resting MCAv in either the pooled exercise group (*r* = −0.155, *p* = 0.376) or the control group (*r* = −0.249, *p* = 0.304). No significant correlations were detected between RBANS, MAC-Q, or TMT-B and other cerebrovascular functional outcomes (i.e., resting PCAv, visual NVC, cerebrovascular CO_2_ reactivity, or dynamic cerebral autoregulation).

## 4 Discussion

Our study was based on the premise that brain function and cerebral blood flow are closely linked in humans (Ogoh, 2017). It is well established that stimuli increasing neural activity also increase cerebral blood flow (Attwell et al., 2010), suggesting that repeated episodic increases in blood flow, such as those induced by exercise training, may promote adaptations in cerebrovascular and cognitive function (Walsh et al., 2003). In the present study, cerebrovascular measures primarily reflect MCAv using transcranial Doppler.

Our findings indicate that individual improvements in neuropsychological status (RBANS) and self-perceived memory (MAC-Q) were associated with reductions in resting MCAv in otherwise healthy participants with subjective memory complaints. These associations occurred in the absence of significant between-group differences in cognitive outcomes, except for TMT-B improvements in the WW group, suggesting that the observed relationships reflect within-individual adaptations rather than a robust intervention effect. No such relationship was observed in control participants.

If replicated, these findings may suggest that individuals who exhibit greater cerebrovascular adaptations to exercise also tend to demonstrate greater improvements in selected cognitive outcomes. The observed associations could be explained by exercise-related changes in cerebrovascular reserve or neurovascular efficiency, although neither mechanism was directly assessed in the present study. Consistent with this, higher cardiorespiratory fitness has been associated with lower oxygen extraction fraction, suggesting improved metabolic efficiency despite variable changes in cerebral perfusion (Sanami et al., 2026). Such adaptations may reflect more efficient visual NVC or reduced metabolic cost of neural activity, in line with established relationships between neural activity and cerebral blood flow (Walsh et al., 2003; Attwell et al., 2010; Ogoh, 2017), and known exercise-induced vascular adaptations (Gertz et al., 2006; Green et al., 2017; Barnes and Corkery, 2018; Chong et al., 2018; Green and Smith, 2018; Song et al., 2025). Given the number of outcomes assessed, these findings should be interpreted as exploratory and require confirmation in larger studies.

The interpretation of changes in self-perceived memory warrants caution. Reductions in subjective memory complaints may reflect decreased distress and do not necessarily correspond with objective cognitive performance (Reid et al., 2012). Subjective and objective measures capture related but distinct constructs and may change independently. In the present study, relationships between MAC-Q scores and objective memory measures (e.g., RBANS indices), or mood (e.g., HADS) were not examined. As such, it remains unclear whether changes in perceived memory reflect objective cognitive improvement, affective changes, or both. The differing associations observed between MCAv and objective versus subjective outcomes further suggests partially distinct underlying mechanisms. Future studies should directly examine these relationships.

Several vasoactive and signalling pathways may underlie the relationship between exercise, cerebral blood flow, and cognition. For example, nitric oxide bioavailability increases with exercise (Green et al., 2017) and is implicated in neurogenesis (Chong et al., 2018). In this study, MCAv was used as a surrogate of cerebrometabolic demand, although it is influenced by multiple physiological factors and does not directly measure cerebral metabolism. The impacts of exercise and vascular function have been shown to be particularly potent on certain cognitive domains (Bliss et al., 2021). Cognitive domains assessed were selected to capture multidomain function (memory, attention, and executive function), which are sensitive to early cognitive change, and are known to respond to exercise (Barnes and Corkery, 2018). These domains are also particularly vulnerable to vascular dysfunction, especially executive function and processing speed (O’Brien et al., 2003), although the latter was not assessed in isolation. Overall, our findings are consistent with the hypothesis that individual differences in cognitive responses to exercise may be related to differences in cerebrovascular adaptation; however, the concept of enhanced “cerebral efficiency” remains speculative and requires direct investigation.

Previous studies examining age-related changes in cerebral blood flow have often relied on cross-sectional designs, although longitudinal work is emerging (Snow et al., 2026). Similarly, relationships between fitness, cerebrovascular function, and cognition have frequently been inferred from between-participant comparisons, which are subject to confounding factors such as body composition and cardiovascular status (Poels et al., 2008). Randomised controlled trials, including the present study, reduce these limitations by enabling within-participant comparisons. Such studies suggest that exercise may improve cerebrovascular function (Murrell et al., 2013), although findings are mixed and likely population- and exercise modality-dependent. In this cohort, we previously reported modality-specific effects on cerebrovascular measures, including CO_2_ reactivity, visual NVC, and autoregulation, and observed that greater improvements in cardiorespiratory fitness were associated with a decrease in MCAv hypercapnic reactivity following exercise training (Green et al., 2021).

The current study extends these findings by examining relationships between exercise-induced changes in cerebrovascular and cognitive outcomes. While exercise is widely recognised as beneficial for cognition in ageing populations (Livingston et al., 2020), relatively few studies have assessed these outcomes concurrently. One study reported associations between cognitive changes and cerebrovascular reactivity during hypercapnia (Guadagni et al., 2020), whereas we observed associations between reduced resting MCAv and improved cognition, suggesting different underlying mechanisms. It has been proposed that early stages of cognitive decline may involve compensatory increases in cerebral blood flow. Within this framework, the reductions in MCAv observed here could be interpreted as being consistent with a normalisation of this response; however, this explanation remains speculative. Accordingly, our findings are not directly comparable to studies examining cerebrovascular reactivity or hypercapnic responses. Adherence to the intervention was high (~80%), and training intensity was comparable between groups (Green et al., 2018b; Naylor et al., 2020), suggesting that the lack of between-group effects is unlikely due to insufficient stimulus. A group-specific improvement in executive function was observed for TMT-B in the WW group; however, this isolated finding should be interpreted cautiously. Overall, larger randomised controlled trials are needed to further examine relationships between cerebrovascular and cognitive adaptations.

At face value, these findings may appear inconsistent with literature linking reduced cerebral perfusion to cognitive impairment. However, compensatory mechanisms such as cognitive reserve, neural recruitment, and network reorganisation may allow cognition to be maintained despite lower blood flow. Our findings may therefore be compatible with the hypothesis of improved cerebral efficiency, whereby cognitive function is maintained at lower metabolic demand; however, this interpretation cannot be confirmed from the present data. This interpretation aligns with evidence that higher fitness is associated with lower resting MCAv and reduced grey matter perfusion (Furby et al., 2020), and that exercise effects may be restorative rather than augmentative in relatively healthy populations (Kaiser et al., 2022). However, it is important to note that transcranial Doppler does not assess vessel diameter, and regional changes in cerebral perfusion may not be captured by global measures such as MCAv (Alfini et al., 2016).

This study has several limitations. It was not powered for cognitive outcomes, and findings should be interpreted cautiously. Participants were relatively healthy older adults with subjective memory complaints, limiting generalisability to more cognitively impaired populations. The sample was predominantly female, and sex-specific differences in vascular function may influence responsiveness to exercise (Moreau et al., 2013; Thomas et al., 2026). Differences in social interaction between intervention and control groups may also have contributed to outcomes, and future studies should consider active control conditions. The internal consistency of the MAC-Q is relatively low (Ibnidris et al., 2022), and MCAv represents an indirect measure of cerebral perfusion influenced by multiple physiological factors. Although most changes in MCAv were modest, variability may reflect inherent measurement error and technical limitations of transcranial Doppler, including probe positioning. The use of fixed inspired CO_2_ may also have influenced cerebral blood flow responses (Coverdale et al., 2014; Hoiland and Ainslie, 2016; Verbree et al., 2017; Hoiland et al., 2019).

In conclusion, improvements in neuropsychological status and subjective memory were associated with reduced resting MCAv in older adults with subjective memory complaints. These exploratory findings suggest that individual variability in cerebrovascular responses to exercise may be related to variability in cognitive responses. Whether changes in cerebrovascular function contribute mechanistically to cognitive adaptations remains unknown and requires confirmation in larger, adequately powered studies.

## Data Availability

The raw data supporting the conclusions of this article will be made available by the authors, without undue reservation.
